# MRI-based porosity index (PI) and suppression ratio (SR) in the tibial cortex show significant differences between normal, osteopenic, and osteoporotic female subjects

**DOI:** 10.3389/fendo.2023.1148345

**Published:** 2023-03-21

**Authors:** Saeed Jerban, Yajun Ma, Dina Moazamian, Jiyo Athertya, Sophia Dwek, Hyungseok Jang, Gina Woods, Christine B. Chung, Eric Y. Chang, Jiang Du

**Affiliations:** ^1^ Department of Radiology, University of California, San Diego, CA, United States; ^2^ Radiology Service, Department of Research, Veterans Affairs San Diego Healthcare System, San Diego, CA, United States; ^3^ Department of Orthopaedic Surgery, University of California, San Diego, CA, United States; ^4^ Department of Medicine, University of California, San Diego, CA, United States

**Keywords:** osteoporosis, cortical bone, MRI, ultrashort echo time (UTE), bone quality

## Abstract

**Introduction:**

Ultrashort echo time (UTE) MRI enables quantitative assessment of cortical bone. The signal ratio in dual-echo UTE imaging, known as porosity index (PI), as well as the signal ratio between UTE and inversion recovery UTE (IR-UTE) imaging, known as the suppression ratio (SR), are two rapid UTE-based bone evaluation techniques developed to reduce the time demand and cost in future clinical studies. The goal of this study was to investigate the performance of PI and SR in detecting bone quality differences between subjects with osteoporosis (OPo), osteopenia (OPe), and normal bone (Normal).

**Methods:**

Tibial midshaft of fourteen OPe (72 ± 6 years old), thirty-one OPo (72 ± 6 years old), and thirty-seven Normal (36 ± 19 years old) subjects were scanned using dual-echo UTE and IR-UTE sequences on a clinical 3T scanner. Measured PI, SR, and bone thickness were compared between OPo, OPe, and normal bone (Normal) subjects using the Kruskal–Wallis test by ranks. Spearman’s rank correlation coefficients were calculated between dual-energy x-ray absorptiometry (DEXA) T-score and UTE-MRI results.

**Results:**

PI was significantly higher in the OPo group compared with the Normal (24.1%) and OPe (16.3%) groups. SR was significantly higher in the OPo group compared with the Normal (41.5%) and OPe (21.8%) groups. SR differences between the OPe and Normal groups were also statistically significant (16.2%). Cortical bone was significantly thinner in the OPo group compared with the Normal (22.0%) and OPe (13.0%) groups. DEXA T-scores in subjects were significantly correlated with PI (R=-0.32), SR (R=-0.50), and bone thickness (R=0.51).

**Discussion:**

PI and SR, as rapid UTE-MRI-based techniques, may be useful tools to detect and monitor bone quality changes, in addition to bone morphology, in individuals affected by osteoporosis.

## 1 Introduction

According to the World Health Organization, bone mineral density (BMD) assessment using dual-energy x-ray absorptiometry (DEXA) is the standard method for osteoporosis (OPo) diagnosis (1–4). Notably, a major portion of bone volume (>55% in cortical bone and >90% in trabecular bone) (5) is comprised of the organic matrix, water, and fat, which cannot be accurately evaluated *via* DEXA measurement or other x-ray-based techniques (6).

An increasing number of musculoskeletal research groups are investigating the potential benefits of utilizing magnetic resonance imaging (MRI) for bone evaluation, particularly for quantifying the water components, organic matrix, and fat content in cortical bone (7–10). MRI-based bone evaluation avoids the potential harmful exposures to ionizing radiation associated with x-ray-based techniques (11–14) and provides the opportunity for simultaneous assessment of the surrounding soft tissues (15, 16).

Although conventional clinical MRI sequences can be used for morphological imaging, they are not capable of quantitative evaluation of bone due to the lack of detectable signals (7–9). Specifically, the detected MR signal intensity of bone depends on several factors, including its apparent transverse relaxation time (T2*), which is very short (11, 12) and cannot be captured by conventional clinical sequences. Notably, T2* of bone is on the order of hundreds of microseconds, while the echo times (TEs) in conventional clinical MRI sequences are typically several milliseconds or longer (11, 17). On the other hand, ultrashort echo time (UTE) MRI sequences have TEs on the order of several to tens of microseconds, which are short enough to detect signal from cortical bone directly and consequently enable quantitative assessment of cortical bone (7–9, 11, 12, 18, 19).

UTE-MRI-based evaluation of bone is partly underutilized due to the high cost and time demands of MRI in general. Several research studies have focused on developing rapid and efficient UTE-MRI-based bone evaluation methods to facilitate clinical translational imaging of bone. The signal ratio calculation in dual-echo UTE imaging (20) and the signal ratio between UTE and inversion recovery UTE (IR-UTE) (21) are two remarkable examples of rapid UTE-based bone evaluation techniques, each of which takes less than 5 minutes. Notably, the required time for such measurements depends on the UTE acquisition techniques, which can be two-dimensional (2D) (using cartesian or radial trajectories) (22, 23) or three-dimensional (3D) (using cartesian, radial, spiral, or cones trajectories) (10). Generally, a 2D UTE sequence is faster than a 3D UTE sequence, and a spiral acquisition is faster than a radial or cartesian acquisition. It should be noted that the signal-to-noise (SNR) is one of the major challenges with UTE bone imaging, particularly in the hip and spine with thin cortex. 3D UTE sequences have the advantage of providing significantly higher SNR efficiency than 2D UTE sequences.

Rajapakse et al. (20) have proposed a dual-echo UTE imaging technique to calculate porosity index (PI), which is the signal ratio between two MRI images, one with UTE (TE < 0.05 ms) and one with TE = 2.2 ms (where bound water signal has decayed to near zero, and pore water and fat signals are in-phase at 3T). The first echo image represents the total detectable signal from bone, including bound water (BW), pore water (PW), and fat. The second echo represents mostly PW and fat signals (no BW signal). Therefore, the signal ratio between the two images is hypothesized to correlate with the pores’ volume (assuming that pores are filled with PW and/or fat) to the total volume. Although this technique does not estimate the absolute PW content or fat content, it can provide an estimation of bone porosity. In original validation studies, PI in a limited number of human cadaveric tibiae has shown significant correlations with porosity measured with micro-computed tomography (µCT) (n=16), donor age (n=16), mechanical compression stiffness performed on whole-cross-section tibial specimens (n=18), and collagen estimation from near-infrared spectroscopy (n=18) (20, 24). Recently, the significant correlations of PI with microstructural and mechanical properties were confirmed using 135 cortical bone strips (25). The feasibility of PI calculation *in vivo* and its reproducibility level was also investigated, with a coefficient of variation of 2.2% and an intraclass correlation coefficient of 0.97 reported (20). In another *in vivo* study, PI has shown a significant direct correlation with the chronic kidney disease stage (n=95) (26). However, the PI performance in distinguishing subjects with OPo has not been investigated yet.

In another attempt to develop rapid UTE-MRI-based techniques for bone assessment, Li et al. have proposed “suppression ratio” (SR) index, defined as the ratio between the bone UTE signal and the UTE signal after long-T2 suppression performed *via* dual-band saturation-prepared UTE (DB-UTE) or IR-UTE (21). It is assumed that the UTE image represents the total detectable signal from bone (BW, PW, and fat), while the IR-UTE image represents only the BW signal. Therefore, higher PW and fat signals may result in higher SR magnitudes, indicating a higher cortical porosity. In previous ex vivo validation studies of a limited number of specimens (n=13), SR demonstrated significant correlations with μCT-based bone porosity and donor age (21). Recently, the significant correlations of PI with microstructural and mechanical properties were confirmed in an investigation with a larger sample size (n=135) (25). The feasibility of SR calculation and its reproducibility level were investigated in previous studies (intraclass correlation coefficient of 0.98) (21) SR from *in vivo* studies (n=72) demonstrated significant correlations with volumetric bone mineral density (vBMD) (R=0.64) and age (R=0.67) in healthy subjects (21). However, the SR performance in distinguishing subjects with OPo is yet to be investigated.

This study aimed to investigate the performance of PI and SR in detecting bone quality differences between female osteopenia (OPe), osteoporosis (OPo), and normal (Normal) subjects.

## 2 Materials and methods

### 2.1 Subject inclusion

A total of 82 female subjects were recruited for MRI scans: 37 with normal bone (Normal group, 36 ± 19 years old), 14 OPe (72 ± 6 years old), and 31 OPo (72 ± 6 years old). The inclusion criteria for each group were as follows: (1) Normal group: pre-menopausal females under 40 years old or post-menopausal females with recent (<one month) DEXA T-scores above -1; (2) OPe group: post-menopausal females with DEXA T-scores between -2.5 and -1; and (3) OPo group: post-menopausal females with DEXA T-scores below -2.5. Subjcets with a history of bone fracture have been excluded from the study. The institutional review board (IRB) of the University of California, San Diego, approved this study, which was conducted in accordance with applicable good clinical practice requirements and the relevant guidelines and regulations. Written informed consent was obtained from each subject.

### 2.2 UTE-MR imaging and data analysis

All subjects were scanned on a 3T MRI (MR750, GE Healthcare Technologies, WI, USA) scanner using an eight-channel knee coil for both RF transmission and signal reception. The imaging slab was centered in the middle of the tibia and localized based on the operator’s experience. The UTE-MRI scans involved: a) dual-echo 3D UTE Cones sequence (repetition time (TR)=100 ms, TE=0.032 and 2.2 ms, flip angle (FA)=10°) for porosity index (PI) measurement (PI= 2^nd^ TE signal divided by UTE signal) (7, 9, 20) and b) 3D adiabatic IR-UTE Cones sequence (TR=100 ms, TI=45 ms, and TE=0.032 ms, FA=20°) to calculate the suppression ratio (SR=UTE signal divided by IR-UTE signal) (7, 9, 21). The field-of-view (FOV), voxel size, in-plane matrix dimension, number of slices, and slice thickness were 140×140×120 mm^3^, 160×160, 0.87×0.87×5 mm^3^, 24, and 5 mm, respectively. The total scan time was approximately 10 mins.

Average PI and SR were calculated within regions of interest (ROIs) covering the entire bone cross-section selected by two experienced MRI readers for measuring PI and SR using a home-developed MATLAB (Mathworks, MA, USA) code. MRI measurements were performed on single slice consistently selected in the middle of the acquired stack of images. Local bone thickness was calculated for each pixel as equal to the diameter of the largest fitted circle within the selected ROI. Bone thickness for each subject was calculated by averaging the local thickness of all bone pixels. Intraclass correlation coefficient (ICC) was calculated for PI and SR between the two readers to investigate their reproducibility.

### 2.3 Statistical analysis

The one-sample Kolmogorov-Smirnov test was performed to determine whether the measured PI and SR were normally distributed for each group. The Kruskal–Wallis test by ranks was used to examine the data differences between the three subject groups (Normal, OPe, and OPo). Spearman’s rank correlation coefficients were calculated between DEXA T-score (51 subjects had DEXA scans) and the UTE-MRI-based bone measures (PI and SR). P-values below 0.05 were considered significant. Statistical analyses were performed using MATLAB codes developed by the authors.

## 3 Results

**Table 1** presents the average and standard deviation (SD) values of PI and SR performed on the tibial bone midshaft of the subjects in the three studied groups (Normal, OPe, and OPo groups). Independent measurements by the two readers were averaged. ICCs between these independent measurements are also presented in **Table 1**. For all MRI parameters, ICCs were higher than 0.95, indicating a high consistency between measurements performed by independent readers.

**Table 1:** Average PI, SR, bone thickness, and T-score values for different groups.

	Normal	OPe	OPo	ICC
**PI (%)**	32.7 ± 4.3	34.8 ± 6.7	40.5 ± 7.2	0.97
**SR**	3.2 ± 0.2	3.7 ± 0.5	4.5 ± 0.8	0.96
**Thickness (mm)**	5.4 ± 0.8	4.8 ± 0.7	4.2 ± 0.5	0.98
**T-score**		-1.74 ± 0.7	-2.58 ± 0.5	

**Figure 1:**
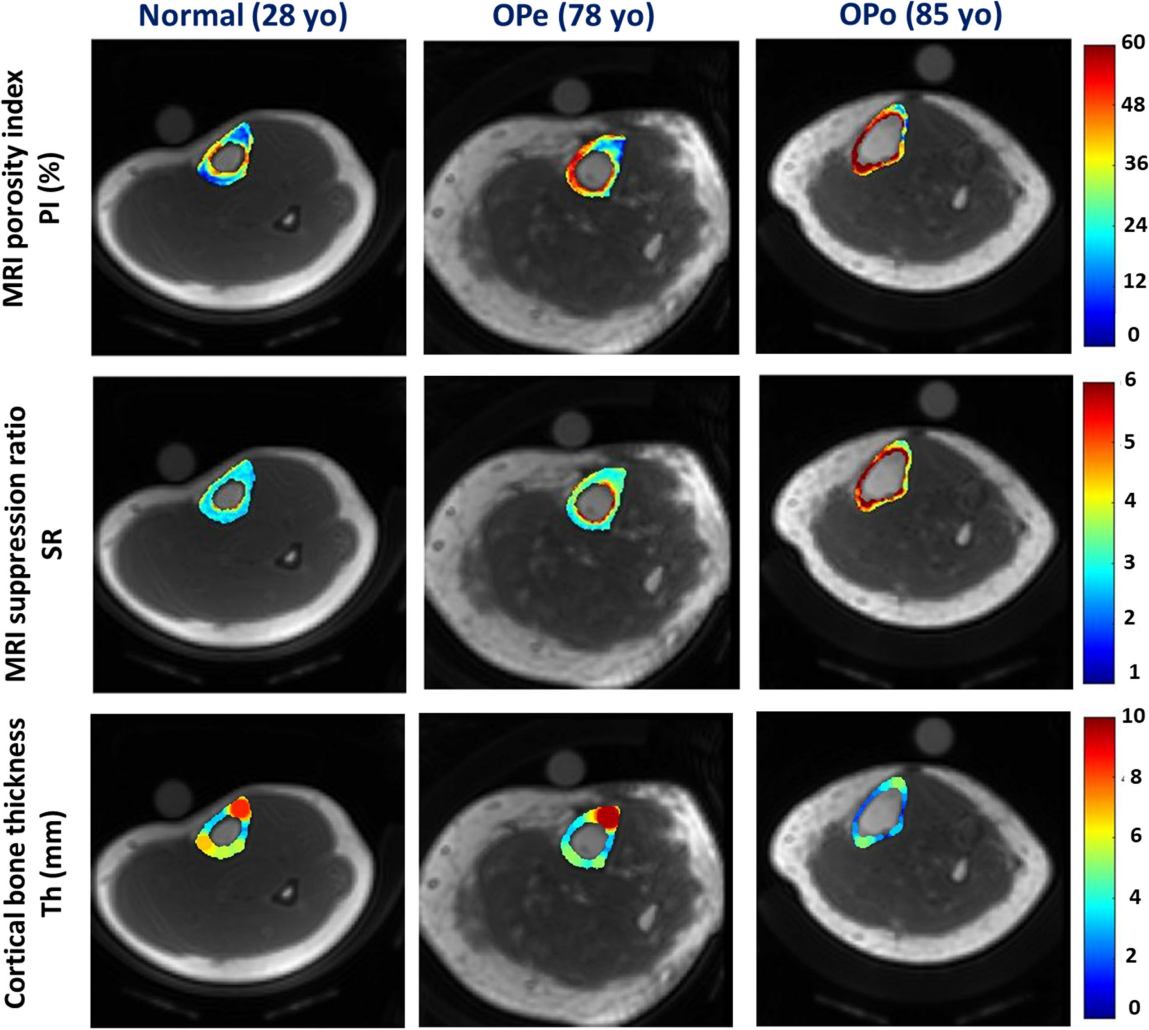
Generated PI, SR, and bone thickness maps for exemplary subjects from the Normal group (first column, 28-year-old female), the OPe group (second column, 78-year-old female), and the OPo group (third column, 85-year-old female). PI and SR were observed in the following ascending order: Normal<OPe<OPo. Regions with higher PI and SR values are likely regions with higher porosity, particularly near the endosteum. In contrast, the mean bone thickness was found in the following descending order: Normal>OPe>OPo.

**Figure 1** demonstrates the generated PI and SR pixel maps for three exemplary subjects from the Normal, OPe, and OPo groups. As expected from **Table 1**, PI and SR values were observed in the following ascending order: Normal<OPe<OPo. In contrast, the mean bone thickness was found in the following descending order: Normal>OPe>OPo.

Percentage differences in PI and SR between the investigated groups and their statistical significance are presented in **Table 2**. PI was significantly higher in the OPo group compared with the Normal (24.1%, p<0.01) and OPe (16.3%, p<0.01) groups. PI in the OPe group was higher than in the Normal group, but the difference was nonsignificant (6.6%, p=0.73). SR was significantly higher in the OPo group compared with the Normal (41.5%, p<0.01) and Ope (21.8%, p=0.02) groups. SR differences between the OPe and Normal groups were also statistically significant (16.2%, p<0.01). Cortical bone was significantly thinner in the OPo group compared with the Normal (22.0%, p<0.01) and OPe (13.0%, p=0.02) groups. Bone thickness in the OPe group was lower than in the Normal group, but the difference did not reach statistical significance (10.3%, p=0.19).

**Table 2:** Percentage difference in PI, SR, bone thickness, and T-score values between the studied groups.

	Normal/OPe	Normal/OPo	OPe/OPo
**PI**	6.6% (p=0.73)	24.1% (p<0.01)	16.3% (p<0.01)
**SR**	16.2% (p<0.01)	41.5% (p<0.01)	21.8% (p=0.02)
**Thickness**	-10.3% (p=0.19)	-22.0% (p<0.01)	-13.0% (p=0.02)
**T-score**			-45.6% (p=0.01)

**Figure 2:**
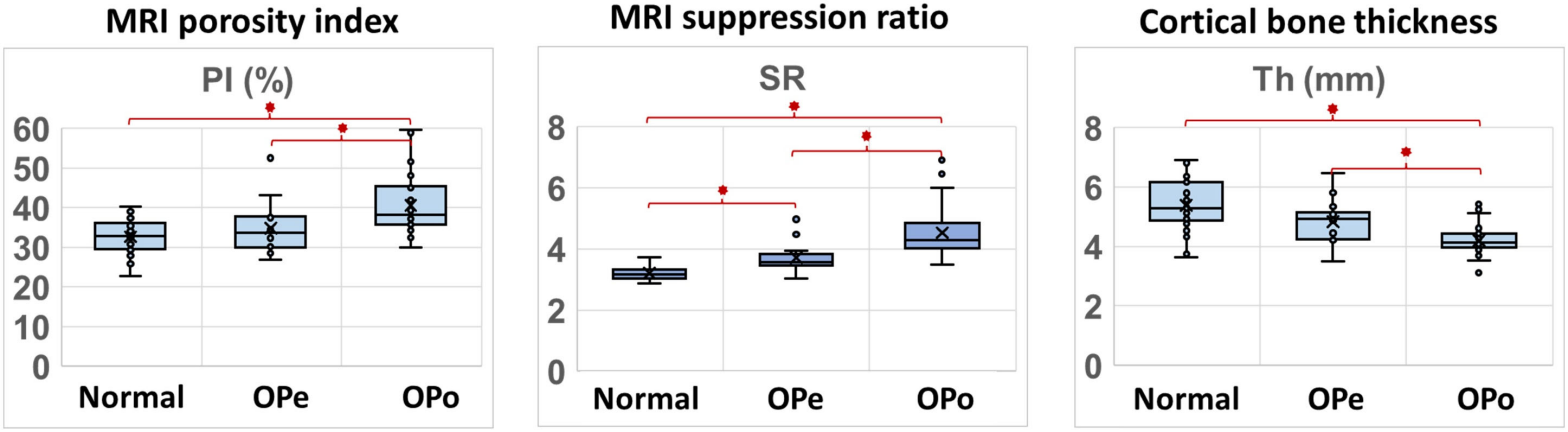
Boxplots of PI, SR, and bone thickness in the Normal, OPe, and OPo groups. Average, median, SD, and first and third quartile values are indicated in the boxplots.

**Figure 2** depicts the average, median, SD, and first and third quartiles of PI, SR, and bone thickness values for each group of subjects using Whisker boxplots. Statistically, significant differences are indicated between groups by horizontal red lines marked with an asterisk.

**Figure 3:**
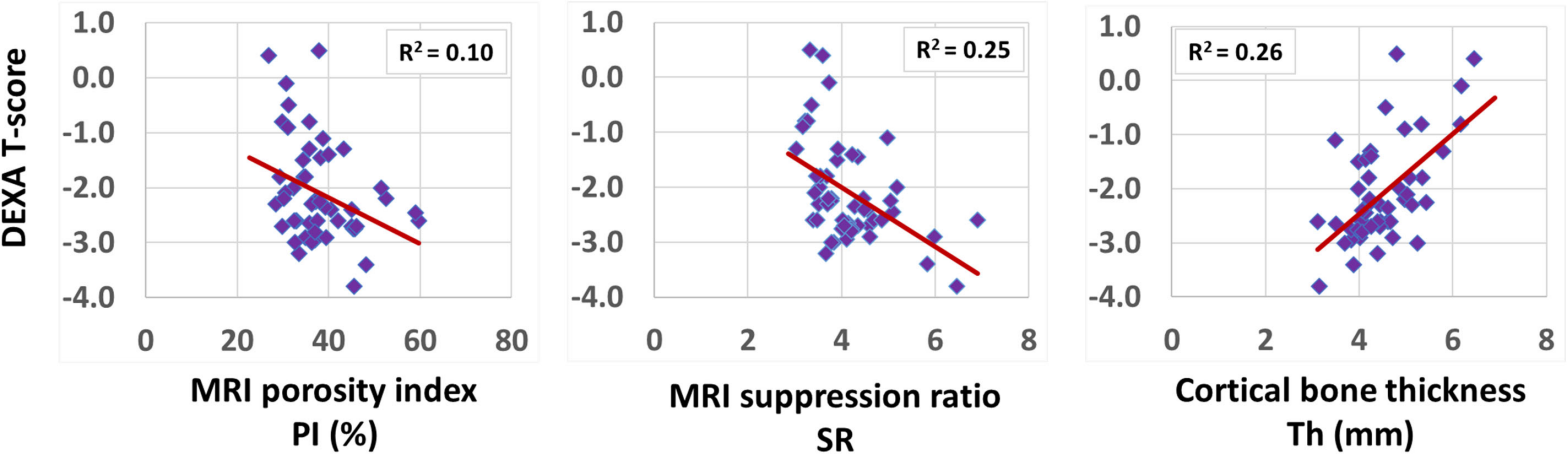
Scatterplots and linear trendlines of DEXA T-score on PI, SR, and bone thickness. R^2^ values were calculated from Spearman’s correlation coefficients.

Spearman’s correlation coefficients between DEXA T-score (performed at the hip) and UTE-MRI measures (performed at the tibial shaft) are presented in **Table 3** (using 51 data points with DEXA scans; young control subjects did not have DEXA scans). SR correlation with T-score was significant (moderate, R=-0.50, p<0.01), while PI showed a significant but poor correlation with T-score (R=-0.32, p<0.01). Bone thickness also showed a significant correlation with T-score (moderate, R=0.51, p<0.01). **Figure 3** demonstrates the scatter plots and the linear regressions of the DEXA T-score on PI, SR, and bone thickness. As expected, higher bone mineral densities are associated with thicker tibial cortex yet lower PI and SR in scanned subjects.

**Table 3:** Spearman’s correlation coefficients between DEXTA T-score and UTE-based measures (PI, SR, and bone thickness).

	PI	SR	Thickness
**DEXA T-Score**	-0.32 (P<0.01)	-0.50 (P<0.01)	0.51 (P<0.01)

## 4 Discussion

This study investigated the differences in PI and SR, two recently developed rapid UTE-MRI-based bone assessment indices, between OPo, OPe, and Normal subjects. These rapid UTE-MRI-based techniques for bone assessment can be considered *in vivo-*translatable techniques due to their simplicity, time efficiency, and, importantly, their non-invasive and ionizing-radiation-free nature.

PI and SR were significantly higher in the studied OPo group compared with the Normal and OPe groups. The SR difference between the OPe and Normal groups was also statistically significant. Higher SR and PI values in OPo subjects can be explained by the anticipated porosity increase in cortical bone during OPo disease development. This study added to the previous feasibility *in vivo* studies of PI and SR, where healthy elderly subjects demonstrated higher PI and SR than young subjects (20, 21). This highlights the potential capability of PI and SR as measures of bone porosity, positioning them as useful and rapid tools for monitoring OPe subjects before OPo advancement, as well as for OPo subjects undergoing medical interventions. It should be noted that the relationships between bone porosity and these UTE-based indices were validated in previous ex vivo studies (20, 21, 25).

A significant moderate correlation was observed between the SR measured at the tibial bone midshaft and the DEXA T-score measured at the hip. PI correlation with T-score was also significant but poor. The reported correlations of PI and SR with vBMD in prior investigations (21), both performed at the tibia, were higher compared to the presented correlation in this study. Although it can be assumed that the bone matrix deterioration occurs across the entire lower extremity at similar rates, higher correlation levels would be expected between PI/SR and DEXA T-score if the same bone sites were investigated in the study. Moreover, it is likely that PI and SR detect the PW signal in slightly different ranges of pores; therefore, they did not demonstrate a similar level of correlation with the DEXA T-Score.

It should be noted that the required scan time for all UTE MRI techniques can be improved by different acceleration techniques such as spokes stretching in Cones (27), compressed sensing (28, 29), and parallel imaging (30, 31). Since both PI and SR measurements require only two acquisitions, they may be faster than other techniques which require multiple acquisitions (19, 32–42), if similar acceleration techniques are utilized. This applies also to other techniques with single or dual acquisitions, such as the PW and BW direct imaging techniques, employed by Horch et al. (43) and Manhard et al. (44), as well as the MTR technique employed by Chang et al. (45).

PI and SR have the potential to monitor the subvoxel cortical bone quantity changes. Subvoxel bone quantifications (e.g., porosity) can play a critical role in determining the bone fracture risk if combined with the current fracture risk assessments. Notably, bone strength is highly determined by the bone structure and its subvoxel material properties. Along with increases in PI and SR in OPo subjecs, cortical bone was significantly thinner compared with the Normal and OPe groups. Similar bone thinning has been reported in previous MRI-based (46) and high-resolution peripheral quantitative CT (HR-pQCT) (47)studies. In light of this fact, comprehensive cortical bone fracture risk evaluation is suggested by complementing the current standard measures (e.g., BMD and FRACS) with bone morphology and MRI-based subvoxel quantity measures.

The limitations of this study can be summarized in five aspects. First, while the presented techniques were translated to *in vivo* applications, only a limited number of subjects were recruited for this study. These techniques must be examined on a larger cohort of OPe and OPo subjects to confirm their clinical applications for OPo disease monitoring. Second, SR magnitude is related to the selection of TR and TI, which was based on our experience with SNR improvement and efficient PW signal nulling. There might be an optimal TR/TI combination that could further improve the performance of SR detecting bone deteriorations in OPo subjects, even though, based on the current parameters, PI and SR demonstrated comparable performance. Third, we have investigated the correlations of PI and SR performed at the tibial midshaft with the DEXA T-scores at the hip. Future *in vivo* validation studies using HR-pQCT or DEXA performed on the tibial midshaft may be required to confirm the significant correlations between our rapid UTE-based indices and bone microstructural changes. Fourth, tibial bone is not the prominent fracture site in most OPo subjects; however, because of the relatively thick cortical bone in tibias, a robust investigation of the UTE-MRI feasibility has been possible. Future investigations should be focused on the hip or spine which present more fracture morbidity and mortality and similarly much more of a challenge for UTE-MRI imaging encountering a thinner bone with sophisticated morphology, located deeper inside the body. Such studies also provide the opportunity for comprehensive comparisons between UTE-MRI and DEXA data which usually are acquired at the hip or spine, such as bone area, BMD, bone mineral content, and bone texture in addition to the often-used T-score. Fifth, PI, SR, and bone thickness were calculated in a single slice in the middle of the tibial shaft. The potential variations of MRI measures across the length of the tibia likely influenced the presented results in this study. Employing an automatic approach for ROI selection in future studies would help to investigate the MRI measurement variations across the entire scanned volumes.

## 5 Conclusion

We investigated the differences in PI and SR, two recently developed rapid UTE-MRI-based bone assessment indices, between OPo, OPe, and Normal subjects. These rapid UTE-MRI-based techniques for bone assessment can be considered *in vivo-*translatable techniques due to their simplicity, time efficiency, and, importantly, their non-invasive and ionizing-radiation-free nature. PI and SR were significantly higher while bone was significantly thinner in the OPo group compared with the Normal and OPe groups. DEXA T-scores in subjects were significantly correlated with PI, SR, and bone thickness. This study highlighted PI and SR as potential rapid UTE-MRI techniques to assess and monitor the quality of cortical bone in patients affected by OPo.

## Data availability statement

The raw data supporting the conclusions of this article will be made available by the authors, without undue reservation.

## Ethics statement

The studies involving human participants were reviewed and approved by University of California, San Diego. The patients/participants provided their written informed consent to participate in this study.

## Author contributions

All authors contributed to the article and approved the submitted version.
